# *LACHESIS* Restricts Gametic Cell Fate in the Female Gametophyte of *Arabidopsis*

**DOI:** 10.1371/journal.pbio.0050047

**Published:** 2007-02-27

**Authors:** Rita Groß-Hardt, Christina Kägi, Nadine Baumann, James M Moore, Ramamurthy Baskar, Wendy B Gagliano, Gerd Jürgens, Ueli Grossniklaus

**Affiliations:** 1 Institute of Plant Biology and Zürich-Basel Plant Science Center, University of Zürich, Zürich, Switzerland; 2 Developmental Genetics, Center for Plant Molecular Biology (ZMBP), University of Tübingen, Tübingen, Germany; 3 Cold Spring Harbor Laboratory, Cold Spring Harbor, New York, United States of America; Oregon State University, United States of America

## Abstract

In flowering plants, the egg and sperm cells form within haploid gametophytes. The female gametophyte of *Arabidopsis* consists of two gametic cells, the egg cell and the central cell, which are flanked by five accessory cells. Both gametic and accessory cells are vital for fertilization; however, the mechanisms that underlie the formation of accessory versus gametic cell fate are unknown. In a screen for regulators of egg cell fate, we isolated the *lachesis (lis)* mutant which forms supernumerary egg cells. In *lis* mutants, accessory cells differentiate gametic cell fate, indicating that *LIS* is involved in a mechanism that prevents accessory cells from adopting gametic cell fate. The temporal and spatial pattern of *LIS* expression suggests that this mechanism is generated in gametic cells. LIS is homologous to the yeast splicing factor PRP4, indicating that components of the splice apparatus participate in cell fate decisions.

## Author Summary

The selection and specification of the egg cell determine the number of eggs produced by an animal or plant, which in turn dictates how many offspring that organism can produce. In most higher plants, the egg cell forms in a specialized structure consisting of four different cell types. Two cells, the egg cell and the central cell, are fertilized by sperm cells and develop into the embryo proper and the nutritive tissue (endosperm), respectively. These two gametic cells are flanked by accessory cells; but why do some cells become gametic while others differentiate into accessory cells? To answer this question, we looked for mutants in which this process is disturbed. In the *lachesis* mutant, accessory cells become extra egg cells. Interestingly, it seems that the misspecification of these accessory cells results from defects in the gametic cells. This suggests that accessory cells monitor the state of the gametic cells to act as a backup if required, ensuring the formation of the key reproductive cells.

## Abstract 3

In plant egg cells, gametophytes differentiate into both gametic and accessory cells; here the authors characterize a mutant that turns accessory cells into gametic cells.

## Introduction

**Figure 1:**
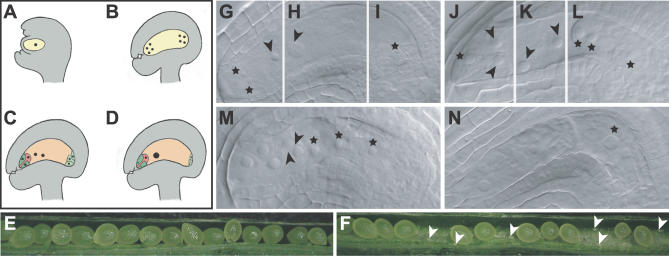
Accessory Cells in *lis-1* Gametophytes Morphologically Resemble Gametic Cells (A–D) Schematic representation of wild-type female gametophyte development. Sporophytic structures are shown in grey; gametophytic structures are colored. (A) After meiosis, the haploid functional megaspore is formed. (B) A series of three mitotic divisions results in the formation of an eight-nucleate syncytium. (C) After nuclear migration and cellularization, a seven-celled gametophyte is formed containing two synergids at the micropylar end (dark green), one egg cell (red), one central cell (orange) with two polar nuclei, and three antipodal cells at the chalazal pole (light green). (D) Prior to fertilization, the two polar nuclei fuse to form one large central cell nucleus, and the antipodal cells degenerate. (E) Wild-type silique showing full seed set. (F) Silique of *lis-1/LIS* plants containing aborted ovules (arrowheads). (G–I) Mature wild-type gametophyte. (G) At the micropylar end, the two small synergid nuclei are detected (stars). The larger egg cell nucleus (arrowhead) is oriented towards the adjacent central cell. (H) A large central cell nucleus (arrowhead) resulting from the fusion of the two polar nuclei can be detected. (I) The antipodal cells at the chalazal end degenerate (star). (J–N) *lis-1* gametophytes. (J) The synergid nuclei are enlarged and mis-polarized (star). As a consequence, synergid and egg cell become indistinguishable when lying in a similar position (arrowheads). (K) Polar nuclei are unfused (arrowheads) and occasionally ectopically cellularized (arrowheads in M). (L and M) Antipodal cells do not degenerate, but enlarge and protrude towards the center (stars). (N) Disintegration of antipodal cells and fused antipodal nuclei (star).

The formation of gametes is a key step in the lifecycle of any sexually reproducing organism. In flowering plants, the egg and sperm cells develop within haploid gametophytes (Figure 1). The female gametophyte of *Arabidopsis* originates from a single haploid spore (Figure 1A) through three nuclear division cycles. The resulting syncytium of eight nuclei (Figure 1B) cellularizes and differentiates four distinct cell fates [1–4] (Figure 1C and 1D). Both the egg and central cells are fertilized by one sperm cell each to form the embryo and the surrounding endosperm, respectively. These gametic cells are flanked by accessory cells. Two synergids lie at the micropylar pole, the entry point of the pollen tube. Synergids are necessary for the attraction of the pollen tube and induce the subsequent release of the sperm cells [5–7]. The opposite pole is occupied by three antipodal cells that degenerate prior to fertilization and whose function is unclear [3].

Although collections of female gametophytic mutants have been reported [8,9], mechanisms that underlie the specification of gametic versus accessory cell fate are unknown. In the present study, we took advantage of an egg cell–specific marker that we isolated in a screen for enhancer detector (ET) and gene trap (GT) lines, to examine the regulation of gametic cell fate. Our results indicate that a combinatorial mechanism operates to ensure maximum likelihood that the key reproductive gametic cells are formed, while at the same time deleterious excess gametic cell formation is prevented.

## Results/Discussion

### In *lachesis-1* Female Gametophytes, All Cells Differentiate Gametic Cell Fate

**Figure 2:**
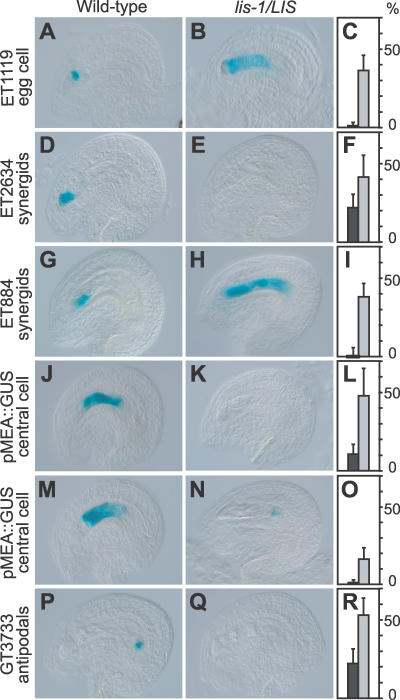
Molecular Analysis of *lis-1* and Wild-Type Gametophytes Expression analysis of cell-specific marker genes in wild-type versus *lis-1*/*LIS* plants (2 d after emasculation if not otherwise indicated). The onset of *GUS* expression for all markers is only detected after cellularization. (A, D, G, J, M, and P) The wild-type expression pattern for each marker. (B, E, H, K, N, and Q) Abnormal expression patterns, representing the scored phenotypes. (C, F, I, L, O, and R) Frequencies of abnormal patterns. Dark bars represent wild-type, light bars represent *lis-1*/*LIS* plants. The *y*-axis shows the percentage of the scored phenotype. (A–C) The egg cell marker ET1119 was expressed ectopically in 36.4% of the gametophytes of *lis-1*/*LIS* plants (*n* = 562; wild-type: 1.2%, *n* = 423). (D–F) The synergid marker ET2634 was not expressed in 41.5% of *lis-1*/*LIS* gametophytes (*n* = 789; wild-type: 22.0%, *n* = 1,176). (G–I) The synergid marker ET884 was expressed ectopically in 38.7% of the gametophytes of *lis-1*/*LIS* plants (*n* = 694; wild-type: 0.1%, *n* = 1,055). (J–L) Expression of the central cell *pMEA::GUS* reporter construct was not detected in 47.8% of the gametophytes of *lis-1*/*LIS* plants (*n* = 596; wild-type: 10.7%, *n* = 428). (M–O) Ectopic expression of *pMEA::GUS* reporter construct in antipodal cells 3 d after emasculation was detected in 16.4% of the gametophytes of *lis-1*/*LIS* plants (*n* = 531; wild-type: 1.3%, *n* = 468). (P–R) The antipodal marker GT3733 was not expressed in 53.1% of the gametophytes of *lis-1/LIS* plants (*n* = 715; wild-type: 22.2%, *n* = 1,283).

We performed a screen for ethyl methanesulfonate (EMS)-induced mutants that alter the expression of the enhancer detector line ET1119, which in wild type confers specific *β-glucuronidase (GUS)* expression to the egg cell (Figure 2A).

In *lachesis-1 (lis-1)* mutants, expression of the egg cell marker was expanded to the synergids and the central cell, suggesting that the restriction of egg cell fate to a single cell is compromised (Figure 2B and 2C). *lis-1* is a loss-of-function mutation for which no homozygous plants were recovered (see below). Therefore, all analyses were performed on heterozygous plants in which only 50% of the ovules contain *lis-1* mutant female gametophytes. Heterozygous *lis-1/LIS* plants produced fertilized seeds and aborted ovules at a 1:1 ratio (50.7%:49.3% in *lis-1/LIS, n* = 631; 96.6%:3.4% in wild type, *n* = 655; Figure 1E and 1F), consistent with a female gametophytic defect [10]. Reciprocal crosses with wild-type plants confirmed that *lis-1* was rarely transmitted maternally (transmission efficiency through the female [TE_F_] = 8.6%, *n* = 347). Paternal transmission was also affected, but less severely (transmission efficiency through the male [TE_M_] = 59.4%, *n* = 367).

**Figure 3:**
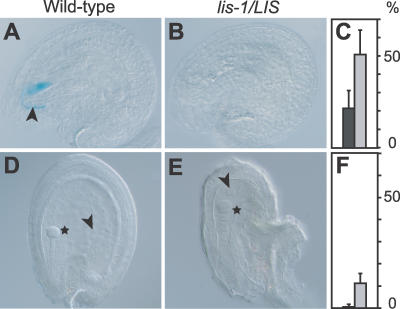
Functional Analysis of Synergids and Central Cells in *lis-1/LIS* Plants (A–C) GUS staining in synergids after fertilization of wild-type and *lis-1/LIS* plants with pollen from the ET434G pollen-tube marker line. (A) Ovule with GUS-stained synergids. The arrowhead points at pollen tube. (B) Ovule in which no GUS staining was detected in synergids. (C) Frequencies of GUS negative synergids. Dark bars represent wild-type, light bars represent *lis-1*/*LIS* plants. The *y*-axis shows the percentage of the scored phenotype (*lis-1/LIS:* 50.8%, *n* = 789; wild-type: 21.5%, *n* = 287). (D–F) Endosperm development after fertilization of wild-type and *lis-1/LIS* plants with wild-type pollen. (D) Ovule with developing embryo (star) and endosperm (arrowhead). (E) Ovule with a developing embryo (star), but no endosperm. The undeveloped central cell nucleus is visible (arrowhead). (F) Frequencies of ovules with a developing embryo, but no endosperm. Dark bars represent wild-type, light bars represent *lis-1/LIS* plants. The *y*-axis shows the percentage of the scored phenotype (*lis-1/LIS:* 11.2%, *n* = 267; wild type: 0.5%, *n* = 191).

To determine whether *lis-1* female gametophytes are indeed defective in cell specification, we examined morphological, molecular, and functional characteristics of the different cell types in *lis-1* gametophytes (Figures 1, 2, and 3, respectively). Until cellularization, gametophytes were indistinguishable between *lis-1/LIS* and wild-type plants (unpublished data), indicating that *LIS* is not required for any previous step, including mitotic divisions, migration of nuclei, or cellularization. The first defects in *lis-1* female gametophytes were consistently observed only after cellularization, corresponding to the wild-type stage at which the different cell types establish distinct morphological and molecular characteristics, as described below. Wild-type synergids differ morphologically from egg cells by two features. First, the synergid nuclei are smaller than the egg cell nucleus. Second, the polarity of synergids is reversed with respect to nuclear position [11,12] (Figure 1G). In *lis-1/LIS* plants, however, synergids differentiated the morphological attributes of egg cell fate and were often indistinguishable from egg cells (Figure 1J; Table 1). Additionally, the expression of the synergid marker ET2634 was down-regulated in many *lis-1* gametophytes (Figure 2D–2F). To test whether the pollen tube–attracting activity of synergids was affected as well, we pollinated *lis-1/LIS* and wild-type plants with a marker line expressing *GUS* in the pollen tube (ET434G) (Figure 3A). The number of ovules without GUS staining was strongly increased in *lis-1/LIS* plants as compared to wild type (Figure 3A–3C), implying that pollen tube attraction was compromised in the majority of *lis-1* mutant female gametophytes. These data, together with the ectopic expression of the egg cell marker in the synergids, indicate that *lis-1* mutant synergids differentiate egg cell attributes at the expense of synergid cell fate. A different synergid marker (ET884) was ectopically expressed in *lis-1* gametophytes (Figure 2G–2I). This intriguing expression shows that not all aspects of accessory and gametic cell fate are mutually exclusive. However, the reduced pollen tube attraction in *lis-1* indicates that this marker is unlikely to reflect fully differentiated synergid cell fate.

**Table 1:**
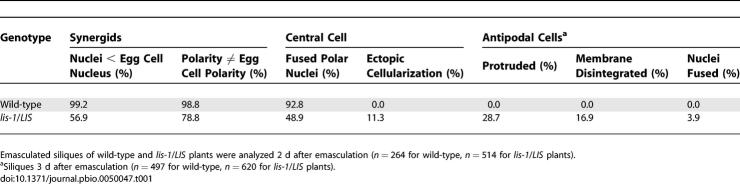
Morphological Analysis of *lis-1/LIS* Plants

The central cell differs from the egg cell by both its size and the presence of two polar nuclei, which fuse prior to fertilization [1–4] (Figure 1H). In *lis-1* mutant gametophytes, the polar nuclei rarely fused and often cellularized separately, a process never observed in wild type (Figure 1K and 1M; Table 1). The resulting uninucleate cells were morphologically indistinguishable from an egg cell. These morphological changes were reflected by the down-regulation of a central cell marker *pMEA::GUS* in most *lis-1* gametophytes (Figure 2J–2L). Whereas the wild-type central cell develops into endosperm after fertilization, approximately one half of the *lis-1* mutant gametophytes that received a pollen tube (compare Figure 3C) failed to develop endosperm (Figure 3D–3F) although embryo formation was not affected. The failure to develop endosperm is unlikely to be related to an unfertilized central cell, because embryo formation can initiate autonomous endosperm formation in an unfertilized central cell [13]. Together with the ectopic expression of the egg cell marker, our results indicate that in *lis-1* gametophytes, the central cell differentiates egg cell attributes at the expense of central cell fate.

A further striking phenotype was observed for the antipodal cells that degenerate prior to fertilization in wild type (Figure 1I). In *lis-1* female gametophytes, the antipodal cells were often enlarged and protruded into the center (Figure 1L and 1M; Table 1). Additionally, in about one third of *lis-1* gametophytes (16.9% in *lis-1/LIS*), the enlarged antipodal cells eventually disintegrated their cell membranes, allowing the fusion of antipodal nuclei into one large nucleus (Figure 1N). In wild-type gametophytes, fusion of nuclei was only detected in central cells (Table 1). Consistently, we observed ectopic expression of the central cell marker *pMEA::GUS* in the protruding antipodal cells (Figure 2M–2O), whereas the expression of GT3733, an antipodal marker line, was down-regulated (Figure 2P–2R). Thus, antipodal cells in *lis-1* mutant female gametophytes can adopt a central cell fate. Remarkably, both the central cells and antipodal cells changed not only their molecular profile according to the newly adopted cell fate, but they also adjusted their nuclear status (uninucleate versus binucleate) accordingly. The originally binucleate central cell cellularized ectopically, resulting in two uninucleate cells, whereas the uninucleate antipodal cells fused, producing a binucleate cell. These findings suggest that an intracellular signaling mechanism senses the number of nuclei in a given cell, and reveal a tremendous, previously unrecognized plasticity of the female gametophyte. In summary, whereas wild-type gametophytes differentiate accessory and gametic cell types, accessory cells of *lis-1* mutant gametophytes frequently adopted gametic cell fate. These observations suggest that all cells in the female gametophyte are competent to differentiate gametic cell fate and that *LIS* is involved in a mechanism that represses gametic cell fate in the accessory cells. Interestingly, the gametic central cell in *lis-1* gametophytes additionally adopted egg cell fate, suggesting that a further, *LIS*-dependent mechanism suppresses egg cell fate in the central cell. Thus, the *lis-1* mutant phenotype reveals two levels of cell fate regulation, one between gametic and accessory cells, and one between egg and central cell.

### Successive Recruitment of Cells as Gametic Cells in *lis-1* Gametophytes

**Figure 4:**
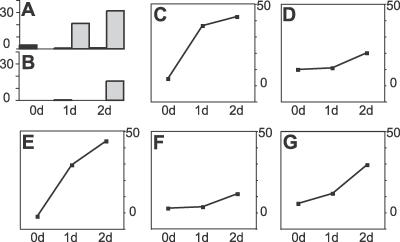
Successive Recruitment of Cells as Gametic Cells in *lis-1* Gametophytes (A and B) Ectopic expression of the egg cell marker ET1119 (A) and the central cell marker *pMEA::GUS* (B) in wild-type and *lis-1/LIS* plants. Dark bars represent wild-type, light bars represent *lis-1*/*LIS* plants. The *y*-axis shows the percentage of ectopic expression of total GUS-staining ovules. Ectopic expression was scored zero (0d), one (1d), and two days (2d) after emasculation. Total number of ovules counted was greater than 250. (C–G) Development of five morphological features in *lis-1/LIS* plants as compared to wild type, zero (0d), one (1d), and two days (2d) after emasculation. The *y*-axis shows percent deviation from wild type (data from Table S1). (C) Synergid nuclei smaller than egg cell nucleus. (D) Different polarity of synergids and egg cell with respect to position of nucleus. (E) Polar nuclei unfused. (F) Ectopic cellularization. (G) Protruded antipodal cells.

The late initiation of cell-specific marker genes indicates that in *Arabidopsis* distinct cell fates are only manifested after cellularization. Studies in several multicellular systems have shown that cell specification is often preceded by the asymmetric distribution of fate determinants (for review see [14]), and an analogous mechanism could be defective in *lis-1* gametophytes, resulting in an instant misspecification of accessory cells. Alternatively, the *lis-1* phenotype could result from defects that occur after cellularization when distinct cell fates become manifest. We analyzed the time course of egg cell and central cell marker gene expression. Interestingly, we found that the number of ovules that ectopically expressed gametic cell fate increased over time (Figure 4A and 4B), indicating that accessory cells are not instantly misspecified as gametic cells. In line with a successive misspecification of accessory cells, we found that several morphological features that distinguish *lis-1/LIS* plants from wild type became more pronounced over time (Figure 4C–4G). Our results suggest that during cell specification in *lis-1* mutant gametophytes, accessory cells become gradually recruited as gametic cells.

### LIS Is Homologous to the Yeast Splicing Factor PRP4

**Figure 5:**
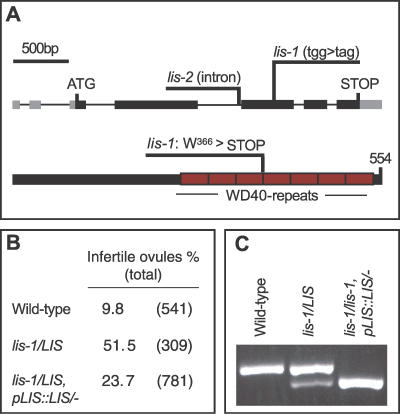
*LIS* Codes for a WD40 Repeat Protein (A) Gene (upper panel) and protein (lower panel) structure of *LIS*. The localization of the *lis-1* and *lis-2* mutations and the seven WD40 repeats are indicated. (B and C) *LIS* cDNA driven by a 2.6-kb upstream promoter complements the gametophytic (B) as well as possible sporophytic defects of *lis-1*/*lis-1* mutants, allowing for the generation of homozygous plants, as demonstrated by PCR-based genotyping (C).

We mapped the *lis-1* mutation to the At2g41500 locus, which encodes a protein with seven WD40 repeats. The *lis-1* mutation creates an in-frame stop codon after three WD40 repeats (Figure 5A). The *LIS* cDNA driven by a 2.6-kilobase (kb) upstream promoter sequence complemented the *lis* mutant phenotype (Figure 5B and 5C), indicating that *lis-1* is a loss-of-function mutation.

The LIS protein is strongly conserved among eukaryotes [15] (Figure S1), showing an overall similarity to *Homo sapiens, Caenorhabditis elegans,* and Saccharomyces cerevisiae of 62%, 56%, and 54%, respectively. The yeast homolog PRP4 is associated with the U4/U6 complex of the spliceosome [16,17]. PRP4 is an essential splicing factor, and loss-of-function mutants accumulate unspliced pre-mRNA [18]. PRP4 function depends on its interaction with a second splicing factor, PRP3, through its WD40 domain, and the deletion of two WD40 repeats abolishes this interaction [19]. We thus conclude that *lis-1* represents the null phenotype, which is consistent with the observation that the *lis-2* T-DNA insertion allele (Figure 5A) causes a very similar phenotype (Figure S2).

The *Arabidopsis* genome contains a second *LIS*-related sequence, At2g05720. The deduced protein (accession no. AAD25639; Figure S1) shares an overall similarity of 70%, but is only half the size of the LIS protein and notably contains only four complete WD40 repeats (Figure S1). Hence, At2g05720 is unlikely to be functionally redundant to LIS.

### *LIS* Is Strongly Up-Regulated in Gametic Cells

**Figure 6:**
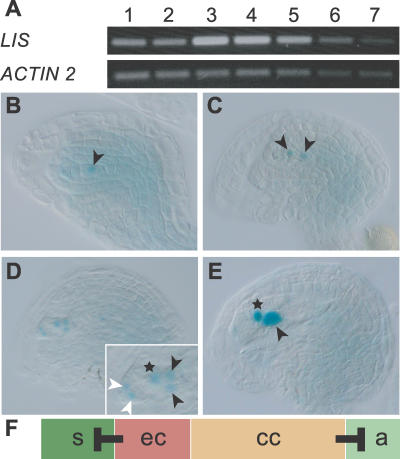
*LIS* Is Strongly Expressed in Gametic Cells (A) RT-PCR analysis of *LIS* expression in leaves (1), roots (2), flower buds (3), open flowers (4), inflorescences (5), siliques (6), and stem (7) (upper panel). *ACTIN 2* was used as control (lower panel). (B–E), Expression of *pLIS::NLS_GUS* in wild-type ovules during female gametophyte development. (B) Arrowhead points at the functional megaspore in which *GUS* expression is detected in the nucleus. (C) Two-nucleate embryo sac showing *GUS* expression in both nuclei (arrowheads). (D) Young eight-nucleate gametophyte. Antipodal nuclei are not visible. Inset shows area with GUS-positive nuclei at higher magnification. White arrowheads point to the synergid nuclei; star points to the egg cell nucleus; and black arrowheads point to the unfused polar nuclei. (E) Mature gametophyte showing strong GUS signal in both the egg cell (star) and fused central cell nucleus (arrowhead). Expression in synergid cells is hardly detectable. (F) Proposed model for *LIS* function. Expression of *LIS* in gametic cells (egg cell [ec] and central cell [cc]) is necessary for the generation of a lateral inhibition signal that prevents the adjacent accessory cells (synergids [s] and antipodal cells [a]) from adopting gametic cell fate.

To determine the temporal and spatial expression pattern of the *LIS* gene, we performed RT-PCR and analyzed the expression of a *pLIS::NLS_GUS* construct containing the same *LIS* promoter fragment as the *LIS* cDNA rescue construct that had fully complemented the mutant phenotype (Figure 5B and 5C). *LIS* expression was detected at moderate levels in all tissues examined, with strongest expression in reproductive tissues (Figure 6A). *GUS* expression driven by the *LIS* promoter was detected at all stages of female gametophyte development (Figure 6B–6E). Intriguingly, shortly after cellularization, expression in the accessory cells is down-regulated, whereas expression in the gametic cells is strongly up-regulated (Figure 6E). This suggests that the mechanism, which prevents accessory cells from adopting gametic cell fate, is not cell-autonomous, but is generated in gametic cells, which is consistent with a lateral inhibition model (Figure 6F). We propose that the gametic cells upon differentiation generate a *LIS*-dependent signaling molecule that is transmitted to the adjacent accessory cells to inhibit their gametic cell competence, thereby preventing excess gametic cell formation. In this view, the *lis* mutant phenotype is a result of impaired lateral inhibition, i.e., the gametic cells fail to identify themselves to their neighboring cells, resulting in the recruitment of all gametophytic cells as gametic cells. The observation that not all cells are synchronously specified as gametic cells implies some initial bias and suggests that the proposed lateral inhibition operates to maintain rather than to establish different cell fates.

Although this mechanism can perpetuate a binary decision between gametic and accessory cell fate, additional factors are needed to explain the generation of four distinct cell fates.

The surprising nature of the LIS protein as a splicing factor suggests the participation of components of the splicing machinery in cell fate decisions and, potentially, the generation of a lateral inhibition signal. A possible mode of action could be that LIS is involved in splicing this very signal or, much less direct, some upstream regulator.

### Conclusion

Our data suggest that a combinatorial mechanism operates to pattern the female gametophyte: The competence of all cells to differentiate gametic cell fate, together with lateral inhibition from the gametic cells, can ensure maximum likelihood that the key reproductive gametic cells are formed, while at the same time, excess gametic cell formation is prevented. Both the expression of the *LIS* gene and its distinct function in regulating gametic cell fate are surprising, given that LIS is the *Arabidopsis* homolog of yPRP4, an integral part of the U4/U6 complex. In the future, identification of LIS target(s) and functional analyses of other tissue-specific splicing factors [20] will help to clarify the mechanistic role of the spliceosome in the regulation of distinct developmental processes.

## Materials and Methods

### Plant material and growth conditions.

Plants were grown on soil in growth chambers under long-day conditions at 18 °C. Enhancer detector and gene trap lines were generated using the system of Sundaresan and colleagues [21]. (Send requests for ET and GT lines to UG, grossnik@botinst.unizh.ch.) The *lis-1* allele was isolated from ET1119 in the Landsberg *erecta* (Ler) accession after mutagenesis; seeds were mutagenized by incubation in 0.15% EMS for 10 h. A total of 5,200 M1 plants were screened for deviating *GUS* expression in the female gametophyte. The *lis-2* allele (SALK_070009) was obtained from the SALK T-DNA insertion collection (http://signal.salk.edu). For the *pMEA::GUS* reporter construct, a gift from D. Page, a 1.6-kb promoter fragment (upstream of the ATG of *MEDEA*) was cloned into pCAMBIA1381z using EcoRI/NcoI restriction sites.

### Molecular cloning and complementation.

Mapping of *lis-1* was done as described [22] using the polymorphisms annotated by CEREON [23]. The Columbia accession (Col) was used as a crossing partner. *lis-1* was located to Chromosome 2 in an area of 126 kb between the polymorphisms CER448978 and CER446310 on BACs F13H10 and T32G6, respectively. We amplified and sequenced *lis-1/LIS* genomic DNA spanning 15 open reading frames and identified a single heterozygous locus in At2g41500. The *LIS* cDNA was isolated according to the annotated open reading frame from a cDNA library using primer 5′-GATTGAGGATCCATGGAACCCAACAAGGAT-3′ and 5′-GATTGAGGATCCAAACAAAGTTCATTCATTTGC-3′ and cloned into pDRIVE (Qiagen, Hilden, Germany). The cDNA was released as an XhoI/KpnI fragment, and cloned into pMDC134 (M. D. Curtis, unpublished data). Upstream of the cDNA, a Gateway recombination site (Invitrogen, Karlsruhe, Germany) was introduced using the XhoI/SacI sites. The *LIS* promoter region was amplified by PCR from genomic DNA using primers 5′-AAGAAACAGCCAAATAGATAAGCA-3′ and 5′-GTTTCCCTTAAATCCTCAAAAGAAAACACC-3′ and cloned into pENTR 1A (Invitrogen) to generate a Gateway compatible promoter fragment. This promoter fragment was cloned upstream of the *LIS* cDNA via Gateway recombination.

### PCR-based genotyping.

Plants were genotyped for the *lis-1* allele using primers 5′-CTACAAGCTATGACAAGACGTGGAGACT-3′ and 5′-TTTTGCTTGGATACGAGGAGGACCAATGGA-3′. The resulting 264 base pair (bp) fragment was digested with BfmI yielding two fragments of 244 bp and 20 bp from the product of the *lis-1* allele, whereas the wild-type product is not digested.

### Expression analysis.

Total RNA was isolated from wild-type tissues using the E.Z.N.A. Plant RNA Kit (Peqlab Biotechnologie, Erlangen, Germany). Ten micrograms of total RNA were used for mRNA-isolation via Dynabeads mRNA DIRECT Micro Kit (Invitrogen). Superscript II (Invitrogen) was used for reverse transcription. Intron-spanning primers were used for PCR to prevent the amplifica-tion of genomic DNA. *LIS* cDNA was amplified using: 5′-CACTGCCTCATACGACATGAAAGTC-3′ and 5′-ACGAGCTATCTGCTGTGATATCTAGAG-3′. *ACTIN2* was amplified using 5′-CCTGAAAGGAAGTACAGTG-3′ and 5′-CTGTGAACGATTCCTGGAC-3′. To generate the *pLIS::NLS_GUS* construct, the *LIS* promoter region was amplified by PCR from genomic DNA using primer 5′-AAGAAACAGCCAAATAGATAAGCA-3′ and 5′-GTTTCCCTTAAATCCTCAAAAGAAAACACC-3′, and cloned into pDRIVE. The 2.6-kb *LIS* promoter fragment was isolated by BamHI/XhoI digestion, and ligated into the binary vector pGIIBAR-EcoRV/XhoI. A *GUS* construct with N-terminal nuclear localization site [24] was inserted downstream of the promoter.

### Histology and microscopy.

For analysis of mature gametophytes, the oldest closed flower bud of a given inflorescence was emasculated and harvested two days later. Cytochemical staining for GUS activity was performed as described [25], without additional clearing of the tissue prior to observation. Whole-mount mature gametophytes were prepared as described by Yadegari et al. [26]. For whole-mount embryos, siliques were dissected and cleared with chloral hydrate:glycerol:water-solution 8:3:1 (w:v:v) without prior fixation. GUS-stained tissue and cleared whole mounts were visualized using a Zeiss Axioscop Microscope (Zeiss, Oberkochen, Germany).

## Supporting Information

Figure S1 Alignment of LIS, *Arabidopsis* AAD25639, H. sapiens, C. elegans, and S. cerevisiae PRP4 Proteins The alignment was generated using CLUSTALW 1.8 with default parameters and BoxShade 3.21. Positions of identical and similar sequences are boxed in black and grey, respectively. The seven repeats of the WD40 motif as annotated by Horowitz et al. [15] are boxed in red; c indicates *C. elegans,* h indicates *H. sapiens,* and y indicates S. cerevisiae PRP4 proteins.
(1.9 MB PDF)

Figure S2 Expression Analysis of *lis-2* and Wild-Type Gametophytes and Localization of T-DNA Insert (A) Wild-type expression pattern of ET1119.
(B) Abnormal expression pattern in *lis-2*.
(C) Frequencies of ectopic egg cell marker expression. Dark bar represents wild-type, grey bar and white bar represent *lis-1*/*LIS* and *lis-2/LIS* plants, respectively. The *y*-axis shows the percentage of the scored phenotype (13.52% in *lis-2/LIS* plants, deduced from 6.76% expression in *lis-2/LIS, ET1119/+* plants, *n* = 1,021. For details on wild-type and *lis-1/LIS* data, refer to Figure 2A–2C).
(D) T-DNA insertion site and sequence of left border flanking region. Red indicates the T-DNA sequence; grey indicates the intron sequence; and black indicates the exon sequence.
(506 KB PDF)

Table S1 Progression of the Morphological Phenotype in Wild-Type and *lis-1/LIS* Plants (60 KB DOC)

### Accession Numbers

The National Center for Biotechnology Information (http://www.ncbi.nlm.nih.gov) accession numbers for proteins discussed in this paper and the supporting information are cPRP4 (NP_492363), hPRP4 (AAC51925), LIS (AAW80862), LIS homologous sequence (AAD25639), and yPRP4 (P20053).

## Abbreviations

GUS: β-glucuronidase
kb: kilobase
*LIS*: *LACHESIS*

## Note Added in Proof

While this manuscript was in press, Raab and Hoth [27] reported that *LIS/AtPRP4* is repressed by abscisic acid and is required for seed development.
