# Supplementary material for: LACHESIS Restricts Gametic Cell Fate in the Female Gametophyte of Arabidopsis
Source: PLoS Biol. 2007 Feb 27;5(3):e47. doi: 10.1371/journal.pbio.0050047 (PMC1804285; doi:10.1371/journal.pbio.0050047)
Supplement: Supplementary file 1 [file pbio.0050047.sg001.pdf]

```
LIS          1 MEPNKDDNVSLAATAQISAPPVLQDASSLPGFSAIPPVVPPSFPPPMAPIPMMPHPPVAR
AAD25639     1 ------------------------------------------------------------
hPRP4        1 ------------------MASSRASSTQATKTKAPDDLVAPVVKKPHIYYGSLEEKERER
cPRP4        1 ----------------------------------------------MAENGNFAVPAPPR
yPRP4        1 ----------------------------------------------MSKYIALENLPVDL

LIS         61 PPTFRPPVSQNGGVKTSDSDSESDDEHIEISEESKQVRERQ-EKALQDLLVKRRAAAMAV
AAD25639     1 ------------------------------------------------------------
hPRP4       43 LAKGESGILGKDGLKAGIEAGNINITSGEVFEIEERISERQ-AEVLAEFERRKRARQINV
cPRP4       15 QFGSLANAESVNAILNAQQQNHGPTVSLERMEVSNQADSRHDAEMFAEFDRRRRARTLTL
yPRP4       15 QHKGATQNES-------------------------TADILKQLP--HERLQ---AVLEKI

LIS        120 PTNDKAVRDRLRRLGEPITLFGEQEMERRARLTQLLTRYDINGQLDKLVKDHEEDVTPKE
AAD25639     1 ------------------------------------------------------------
hPRP4      102 STDDSEVKACLRALGEPITLFGEGPAERRERLRNILSVVGTD-ALKKTKKDD-----EKS
cPRP4       75 PTDDVQVKLKLRALNQPICLFGEDALDRRERLRALLSTMSED-EIAAVLHTDEVN--ADK
yPRP4       45 PEEDLEVRRLLSILKKPEVVENEDVQQRRIRLAEILMVDEID--LENINNMENIN--GEE

LIS        180 EVDDEVLEYPFFTEGPKELREARIEIAKFSVKRAAVRIQRAKRRRDDPDE-DMDAETKWA
AAD25639     1 -------------MGPTELREARIEITKDFIKRAALRIQRENRRRNDPDE-DKNAETKLA
hPRP4      156 KKSKEEYQQTWYHEGPNSLKVARLWIANYSLPRAMKRLEEARLHKEIPET-TRTSQMQEL
cPRP4      132 ADEET---VTWYHRGPIELRMARVSIADFSLRKAKLRLDKAREEAARPAH-EKALARQEA
yPRP4      101 VDEED--DEDFFTPATSELIFARRFLINYSLERSRKRLQKEMERHQKFNTRQELLSRRTE

LIS        239 LKHAKHMALDCSNFGDDRPLTGCSFSRDGKILATCSLSGVTKLWEMPQVTNTIAVLKDHK
AAD25639    47 LKHCKDMVLGSSKFGDDRPLTGCSLSRDGKILVTCSLSGVPKLWEVPQVTNKIVVLKGHK
hPRP4      215 HKSLRSLNNFCSQIGDDRPISYCHFSPNSKMLATACWSGLCKLWSVPDCN-LLHTLRGHN
cPRP4      188 HKWVQQINLHASQVADTRPVAFCEFSADSEHIVTAGWSGSVAVWKREQCA-QEIKFIGHS
yPRP4      159 LQRMANLELAGSQLVSTKPISAVSLSTDDMVVATGSWAGDLQVLNSQTLQPLTQKLDSHV

LIS        299 ERATDVVFS--------PVDDCLATASADRTAKLWKTDG-----TLLQTFEGHLDRLARV
AAD25639   107 EHVTDVVFSS-------VDDECLATASTDRTEKIWKTDG-----TLLQTFK---------
hPRP4      274 TNVGAIVFHPKSTVSLDPKDVNLASCAADGSVKLWSLDSD----EPVADIEGHTVRVARV
cPRP4      247 SQAGCARFHPGAFTQNDYSSLNVVSCSYDGTVLLWSLSQE----SPIGELEQHPQRVSKV
yPRP4      219 GKIGAIDWHP-------DSNNQMISCAEDGLIKNFQYSNEEGGLRLLGDLVGHERRISDV

LIS        346 AFHPSGKYLGTTSYDKTWRLWDINTGAELLLQEGHSRSVYGIAFQQDGALAASCGLDSLA
AAD25639   146 ---------------------------------------------------ASSGFDSLA
hPRP4      330 MWHPSGRFLGTTCYDRSWRLWDLEAQEEILHQEGHSMGVYDIAFHQDGSLAGTGGLDAFG
cPRP4      303 AFHPNGHHLATACFDSTWRMYDLTTKKELLYQEGHSKSVADVAFHPDGSVALTGGHDCYG
yPRP4      272 KYHPSGKFIGSASHDMTWRLWDASTHQELLLQEGHDKGVFSLSFQCDGSLVCSGGMDSLS

LIS        406 RVWDLRTGRSILVFQGHIKPVFSVNFSPNGYHLASGGEDNQCRIWDLRMRK--SLYIIPA
AAD25639   155 RVWDLRTARNILIFQGHIKQVLSVDFSPNGYHLASGGEDNQCRIWDLRMRK--LLYIIPA
hPRP4      390 RVWDLRTGRCIMFLEGHLKEIYGINFSPNGYHIATGSGDNTCKVWDLRQRR--CVYTIPA
cPRP4      363 RVWDMRTGRCIMFLDGHTKEIHSVEWMPNGYEMITGSSDNSMKVWDLRMRR--NTYTMPA
yPRP4      332 MLWDIRSGSKVMTLAGHSKPIYTVAWSPNGYQVATGGGDGIINVWDIRKRDEGQLNQILA

LIS        464 HANLVSQVKYEP-QEGYFLATASYDMKVNIWSGRDFSLVKSLAGHESKVASLDITADSSC
AAD25639   213 HVNLVSQVKYEP-QERYFLATASHDMNVNIWSGRDFSLVKSLVGHESKVASLDIAVDSSS
hPRP4      448 HQNLVTGVKFEP-IHGNFLLTGAYDNTAKIWTHPGWSPLKTLAGHEGKVMGLDISSDGQL
cPRP4      421 HTSVVTRVRAD--AAGQYLVSASFDCTLKMWSTTGWQPLRQLQGHDTRILCVDISPDGQW
yPRP4      392 HRNIVTQVRFSKEDGGKKLVSCGYDNLINVYSSDTWLKMGSLAGHTDKIISLDISNNSHF

LIS        523 IATVSHDRTIKLWTSSGNDDEDEEKETMDIDL
AAD25639   272 RKCFA---------------------------
hPRP4      507 IATCSYDRTFKLWMAE----------------
cPRP4      479 MCSSAFDRTFKLWAQSDY--------------
yPRP4      452 LVSGGWDRSIKLWN------------------
```

Groß-Hardt *et al.*
Figure S1
