# Supplementary material for: LACHESIS Restricts Gametic Cell Fate in the Female Gametophyte of Arabidopsis
Source: PLoS Biol. 2007 Feb 27;5(3):e47. doi: 10.1371/journal.pbio.0050047 (PMC1804285; doi:10.1371/journal.pbio.0050047)
Supplement: Supplementary file 2 [file pbio.0050047.sg002.pdf]

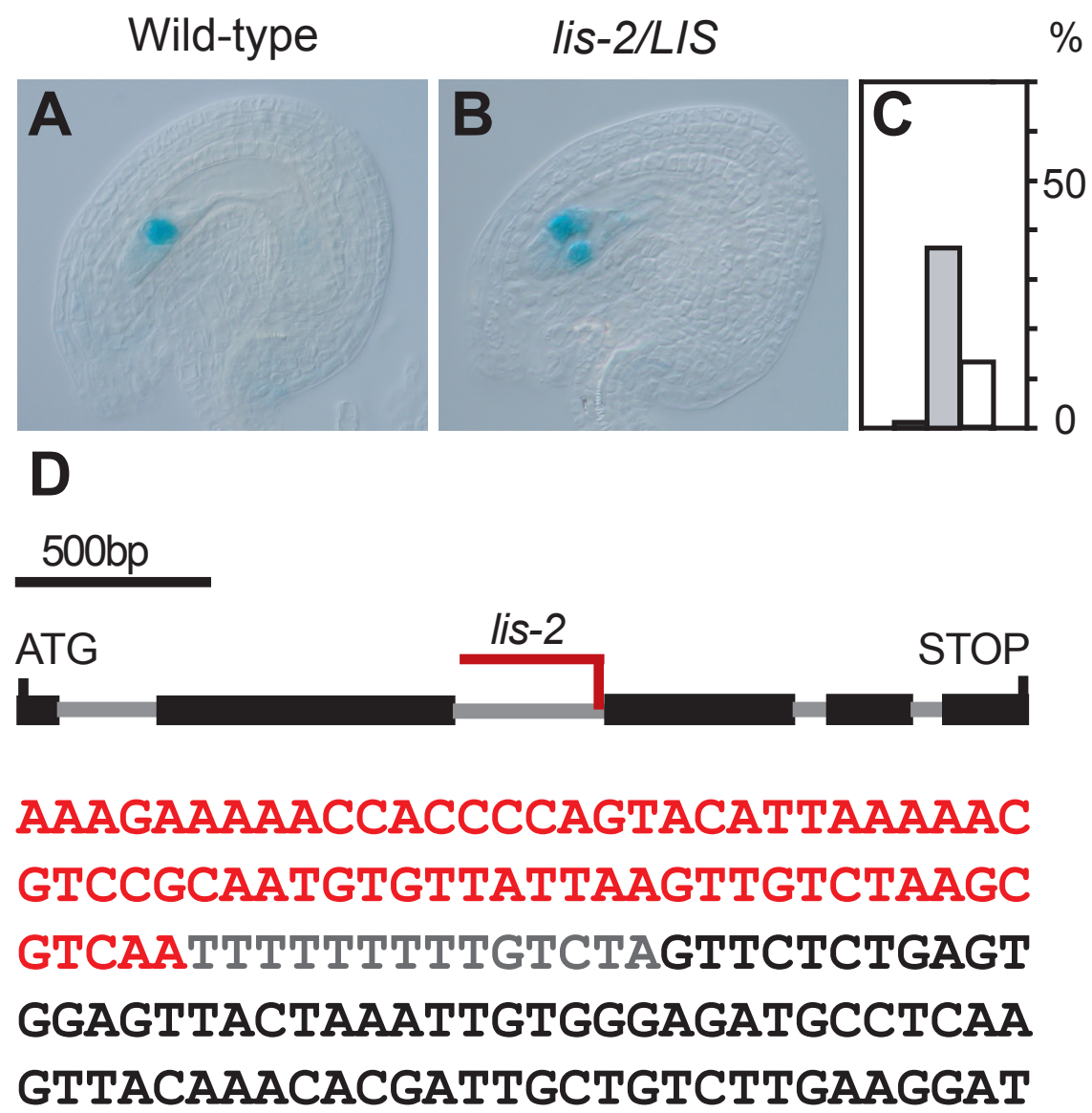

Groß-Hardt *et al.*
Figure S2
