# Supplementary material for: LACHESIS Restricts Gametic Cell Fate in the Female Gametophyte of Arabidopsis
Source: PLoS Biol. 2007 Feb 27;5(3):e47. doi: 10.1371/journal.pbio.0050047 (PMC1804285; doi:10.1371/journal.pbio.0050047)
Supplement: Supplementary file 3 [file pbio.0050047.st001.doc]

Tables

| **Table S1.** Progression of the Morphological Phenotype in Wild-type and *lis-1/LIS* Plants | | | | | | | |
| --- | --- | --- | --- | --- | --- | --- | --- |
|  | **Synergids** | |  | **Central cell** | |  | **Antipodal cells** |
|  | Nuclei <  ec nucleus (%) | Polarity ≠  ec polarity (%) |  | Fused pn (%) | Ectopic cellu-larization (%) |  | Protruded (%) |
|  |  |  |  |  |  |  |  |
| 0d |  |  |  |  |  |  |  |
| Wild-type | 21.7 | 91.2 |  | 26.7 | 0.2 |  | 1.2 |
| *lis-1*/*LIS* | 17.6 | 81.4 |  | 29.1 | 2.7 |  | 6.8 |
|  |  |  |  |  |  |  |  |
| 1d |  |  |  |  |  |  |  |
| Wild-type | 65.5 | 96.9 |  | 78.4 | 0.0 |  | 0.7 |
| *lis-1*/*LIS* | 28.8 | 86.1 |  | 49.2 | 3.4 |  | 12.5 |
|  |  |  |  |  |  |  |  |
| 2d |  |  |  |  |  |  |  |
| Wild-type | 99.2 | 98.9 |  | 92.8 | 0.0 |  | 0.0 |
| *lis-1*/*LIS* | 56.9 | 78.8 |  | 48.9 | 11.3 |  | 29.4 |
| Emasculated siliques of wild-type and *lis-1*/*LIS* plants were analyzed zero, one, and two days after emasculation (0d: n = 409 for wild-type, n = 296 for *lis-1*/*LIS* plants; 1d: n = 417 for wild-type, n = 295 for *lis-1*/*LIS* plants; 2d: n = 264 for wild-type, n = 513 for *lis-1*/*LIS* plants). ec, egg cell; pn, polar nuclei. | | | | | | | |

Groß-Hardt *et al.*

Table S1
